# Spatial transcriptomics uncovers TAC-OGEs heterogeneity and FN1/MMP9 signature in ameloblastoma

**DOI:** 10.3389/fimmu.2026.1770116

**Published:** 2026-04-30

**Authors:** Yitong Wang, Haiyang Li, Chunyu Zhang, Xin Peng, Jianping Liu, Wenya Du, Yao Yu, Yali Hou, Xiangjun Li

**Affiliations:** 1 Department of Oral and Maxillofacial Surgery, School and Hospital of Stomatology, Hebei Medical University, Hebei Technology Innovation Center of Oral Health, Hebei Key Laboratory of Stomatology and Clinical Research Centre for Oral Diseases, Shijiazhuang, Hebei, China; 2 Department of Oral and Maxillofacial Surgery, Peking University School and Hospital of Stomatology, Beijing, China; 3 School and Hospital of Stomatology, Hebei Medical University, Hebei Technology Innovation Center of Oral Health, Shijiazhuang, China; 4 Department of Oral Pathology, School and Hospital of Stomatology, Hebei Medical University, Hebei Technology Innovation Center of Oral Health, Hebei Key Laboratory of Stomatology and Clinical Research Centre for Oral Diseases, Shijiazhuang, Hebei, China

**Keywords:** ameloblastoma, FN1, immunohistochemistry, MMP9, spatial transcriptomics

## Abstract

**Objective:**

This study aims to explore how the tumour microenvironment influences the aggressiveness, malignancy, and recurrence of ameloblastoma. By employing a spatial omics approach, we will examine the characteristics of the invasive front of ameloblastoma.

**Methods:**

We systematically characterised the cellular heterogeneity of ameloblastoma using spatial transcriptomic sequencing combined with integrated omics analysis of clinical tumour specimens. Additionally, we independently validated the spatial expression patterns and levels of FN1 and MMP9 in ameloblastoma through immunohistochemistry in an independent case series of 15 specimens, further supported by single-cell resolution multiplex immunofluorescence.

**Results:**

Spatial transcriptomic sequencing delineated the ameloblastoma tumour ecosystem into seven primary cell clusters: epithelial tumour cells, fibroblasts, myeloid cells, endothelial cells, T cells, B cells, and Tumour-associated cells with osteoclast-like gene expression (TAC-OGEs). Significantly, cell clusters located at the tumour’s invasive front demonstrated notably increased expression of FN1 and MMP9. A more detailed analysis within the TAC-OGE compartment identified seven phenotypically distinct subclusters. Interestingly, their differentiation states formed a spatial gradient, extending from the tumour core to the periphery. This spatial expression pattern of FN1 and MMP9 was confirmed through immunohistochemical staining at the tumour-stroma interface.

**Conclusion:**

Our findings collectively reveal the cellular heterogeneity of ameloblastoma and highlight tumour-margin-associated TAC-OGEs as potential spatially associated components of local invasion. These results suggest that targeting TAC-OGE-associated processes may represent a potential therapeutic strategy in the management of ameloblastoma.

## 1 Introduction

Ameloblastoma (AM) is a benign odontogenic tumour characterised by its local aggressiveness, high recurrence rate, and potential for destructive growth (1). While surgical resection remains the main treatment approach, AM’s infiltrative nature often leads to significant post-operative morbidity. Importantly, the molecular mechanisms driving its local invasive behaviour are not yet fully understood (2).

The tumour microenvironment (TME) is increasingly acknowledged as a crucial factor in ameloblastoma progression. It is proposed that within the TME, extracellular matrix (ECM) remodelling and the induction of epithelial-mesenchymal transition (EMT) work together to facilitate the local invasion of tumour cells (3).

Recent advancements in single-cell RNA sequencing (scRNA-seq) have shed light on the cellular diversity within ameloblastoma, identifying distinct epithelial subpopulations and varied stromal elements (4, 5). Despite these insights, the dissociative nature of scRNA-seq results in a loss of spatial context, hindering our understanding of the spatial organisation and interactions of specific cell types *in situ*. This limitation is especially significant when investigating the role of osteoclasts at the invasive front, which are crucial in orchestrating local tumour progression (6).

The ability to spatially map tumour environments provides a robust means to investigate key mechanisms underlying tumour invasion, notably the epithelial-mesenchymal transition (EMT) (7, 8). In ameloblastoma, EMT characteristics are evident, such as reduced E-cadherin expression alongside increased levels of vimentin and N-cadherin (9, 10). Additionally, essential EMT-inducing transcription factors, including SLUG and ZEB1, are present in ameloblastoma tissues (11). Importantly, in other osteolytic tumours, osteoclasts at the tumour-bone interface are recognised as significant EMT inducers in neighbouring carcinoma cells. This induction largely occurs through the secretion of influential molecules like Transforming Growth Factor-beta (TGF-β) and Matrix Metallopeptidase 9 (12, 13). Drawing parallels from osteotropic malignancies like breast cancer, we hypothesize that osteoclasts at the ameloblastoma-bone interface may similarly serve as significant EMT inducers, although this remains to be definitively proven in odontogenic tumours.

Fibronectin-1 (FN1), a substantial glycoprotein found abundantly in the extracellular matrix, is crucial for cell adhesion, migration, and tissue remodelling (14). In cancer biology, FN1 triggers vital downstream signalling pathways, notably the FAK/SRC and PI3K/AKT pathways. These pathways collectively bolster tumour cell survival, enhance motility, and promote the EMT, thereby expediting metastatic progression across various cancer types (15, 16).

In the realm of ameloblastoma research, bioinformatics analyses have consistently pinpointed FN1 as a key differentially expressed gene, notably exhibiting significant upregulation (17). However, existing studies have largely focused on confirming its expression levels. As a result, the detailed molecular mechanisms of FN1, its upstream regulatory networks, and its spatiotemporal expression dynamics at the invasive front of ameloblastoma remain inadequately explored, highlighting a substantial gap in current understanding.

Matrix Metallopeptidase 9 (MMP9), an essential member of the matrix metalloproteinase family, plays a pivotal role in tumour invasion. It achieves this primarily by degrading crucial ECM components, such as type IV collagen, which compromises the integrity of the basement membrane barrier (18). In addition to structural degradation, MMP9 activity releases latent growth factors, including VEGF and TGF-β, previously sequestered within the ECM. This release not only promotes angiogenesis but also significantly stimulates osteoclast-mediated bone resorption at the tumour’s advancing front (19, 20).

MMP9 plays a pivotal role within a compensatory network of metalloproteinases that regulates osteoclast-mediated bone resorption, contributing to both the initiation and progression of resorptive activities (21). In ameloblastoma, consistently high MMP9 expression has been observed (22). Despite the association, the specific upstream regulatory mechanisms governing MMP9 in ameloblastoma, as well as the spatiotemporal dynamics of its proteolytic activity within the tumour microenvironment, remain unclear. Additionally, the precise role of MMP9 in facilitating the coordinated invasion of ameloblastoma cell nests is yet to be determined. This knowledge gap significantly hinders a full understanding of AM pathogenesis.

In this study, we employed spatial transcriptomics to systematically chart the molecular and cellular landscape of ameloblastoma, identifying a distinct population of “Tumour-associated cells with osteoclast-like gene expression” (TAC-OGEs). Spatial mapping demonstrated that TAC-OGEs are characteristically enriched at the tumour’s invasive front, where, notably, they exhibit a coordinated high expression of FN1 and MMP9, both crucial for ECM remodelling and tumour invasion (15, 21). Intriguingly, high-resolution clustering within the TAC-OGE population revealed seven phenotypically distinct subclusters. These subclusters displayed a notable spatial gradient in their differentiation states, extending from the tumour core to the periphery. Drawing on these integrated findings, we propose a model in which TAC-OGEs at the tumour-stroma interface may contribute to a pro-invasive microenvironment, possibly through the coordinated secretion of FN1 and MMP9. This niche is associated with the invasive phenotype of ameloblastoma, potentially contributing to ECM degradation and enhancing the migratory ability of tumour epithelial cells.

## 2 Article types

Original Research.

## 3 Materials and methods

### 3.1 Sample collection

Tumour specimens for this study, specifically obtained from complete surgical resection tissues, were sourced from the Department of Oral and Maxillofacial Surgery at the Hospital of Stomatology, Hebei Medical University. The fresh tissue specimen was rapidly processed and snap-frozen in OCT compound within 15 minutes post-resection. A representative tumour sample, selected for comprehensive spatial transcriptomic analysis, was taken from a 34-year-old male patient with no prior treatment. This selection was based on the sample’s classic histopathological characteristics and adequate fresh tissue volume. To corroborate the primary findings from the multi-omics analysis, an independent case series of 15 formalin-fixed paraffin-embedded (FFPE) complete surgical ameloblastoma specimens underwent immunohistochemical (IHC) and multiplex immunofluorescence (mIF) validation. The study adhered to the Declaration of Helsinki and received approval from the hospital’s Institutional Review Board (Ethical Approval Number: [2020]005). Written informed consent, specifically permitting the use of tissue for detailed molecular profiling and publication of the results, was obtained from all participants (**Table 1**).

**Table 1:** Clinical characteristics of the ameloblastoma case series for IHC and mIF validation.

Patient ID	Age (years)	Sex	Tumour location	Sample type	Histological subtype
P1	38	M	Mandible,R	Primary	Conventional
P2	67	M	Mandible,R	Primary	Conventional
P3	70	M	Mandible,R	Primary	Conventional
P4	39	F	Mandible,L	Primary	Conventional
P5	15	M	Mandible,R	Primary	Unicystic
P6	27	M	Mandible,L	Primary	Unicystic
P7	24	F	Mandible,R	Primary	Conventional
P8	22	M	Mandible,R	Primary	Unicystic
P9	52	F	Mandible,R	Primary	Conventional
P10	67	M	Mandible,R	Primary	Conventional
P11	24	M	Mandible,L	Primary	Conventional
P12	33	F	Mandible,L	Primary	Unicystic
P13	28	M	Mandible,L	Primary	Conventional
P14	26	F	Mandible,R	Primary	Conventional
P15	20	F	Mandible,L	Primary	Conventional

M, male; F, female; L, left; R, right.

### 3.2 Spatial Transcriptomics (ST)

Frozen ameloblastoma tissue specimens were cryosectioned to a thickness of 10 μm and affixed to Visium Spatial Gene Expression Slides (10x Genomics). Using the standard Visium Spatial Tissue Optimisation protocol, we determined the optimal permeabilisation time for each tissue type to maximise cDNA release. The optimal tissue permeabilisation time was determined to be 18 minutes based on tissue optimization time-course experiments. Library preparation followed the manufacturer’s guidelines using Visium Spatial Gene Expression Reagent Kits. Briefly, tissue sections underwent H&E staining and imaging, followed by permeabilisation, reverse transcription, and second-strand synthesis. Subsequently, cDNA was amplified, and libraries were constructed by adding sample indices and sequencing adapters. Finally, sequencing was conducted on an Illumina NovaSeq 6000 platform, ensuring a minimum of 50,000 reads per spot. The mean sequencing depth was 61,573 reads per spot.

### 3.3 Data processing and clustering

The raw sequencing data, presented as FASTQ files, underwent processing via the Space Ranger pipeline (10x Genomics, v1.3.1). This facilitated alignment to the GRCh38 human reference genome, barcode/UMI counting, and the creation of feature-spot matrices. Subsequent analyses were conducted in R (v4.3.0) using the Seurat (v4.0.0) and SPATA2 (v0.1.0) packages. Quality control measures excluded spots with unique feature counts below 100 or above 6000, as well as those with a high proportion of mitochondrial genes. The SCTransform workflow was employed for data normalisation, scaling, and, where necessary, the integration of multiple samples. Principal component analysis (PCA) was executed, utilising the top 30 principal components for graph-based clustering at a resolution of 0.8 and non-linear dimensionality reduction via UMAP. Through unsupervised clustering, seven distinct cell populations emerged, which were then annotated according to the expression of canonical marker genes specific to each cell type.

### 3.4 Pseudo-temporal trajectory analysis

To explore the differentiation dynamics and state transitions within the heterogeneous TAC-OGE population, we conducted a pseudo-temporal trajectory analysis using Monocle2 (v2.28.0). While not the primary defining DEGs for the overarching cluster, root states were biologically determined based on the localized expression of canonical myeloid/precursor markers to accurately anchor the developmental origin. Subsequently, the order_cells function assigned each cell a pseudotime value. This spatial distribution along the pseudotime axis was visualised and overlaid onto the H&E image, confirming a spatially ordered distribution extending from the tumour centre to the invasive margin.

### 3.5 Cell-cell communication analysis

To deduce potential interactions between identified tumour cell clusters and surrounding stromal and immune cells, we employed the CellChat package (v1.6.1) to analyse cell-cell communication networks. We used the normalised expression data and cell cluster annotations from the Seurat object as inputs. The computeCommunProb function calculated communication probabilities, utilising the curated CellChatDB human ligand-receptor interaction database. The minimum number of cells required in a cluster for cell-cell communication was set to 10, and a p-value threshold of p< 0.05 was applied for significant interactions. Subsequently, we aggregated and analysed the signalling networks at the level of signalling pathways. By comparing differential communication probabilities between the tumour core and invasive front regions, we identified location-specific signalling events.

### 3.6 Differential expression and statistical analysis

To identify differentially expressed genes (DEGs) between clusters within the same sample, such as TAC-OGEs at the margin compared to all other clusters, the FindMarkers function in Seurat was employed, utilising the Wilcoxon Rank Sum test. Genes were deemed statistically significant if they had an adjusted p-value of less than 0.05 and an absolute average log2 fold change greater than 0.25.

### 3.7 Functional enrichment analysis

To elucidate the biological functions and signalling pathways linked to the differentially expressed genes (DEGs), we employed Gene Ontology (GO) and Kyoto Encyclopedia of Genes and Genomes (KEGG) pathway enrichment analyses via the clusterProfiler package (v4.8.2). Terms with an adjusted p-value of less than 0.05 were deemed significantly enriched. Additionally, Gene Set Enrichment Analysis (GSEA) was performed using the fgsea package (v1.26.0) on pre-ranked gene lists, ordered by log2 fold change, to detect more nuanced and coordinated alterations.

### 3.8 Proteomics data source

The existing proteomics dataset used for comparative analysis was previously generated by our research group (23, 24). To ensure full transparency and reproducibility, the detailed raw proteomics profiling data analysed in the current study are provided in **Supplementary Table 1**.

### 3.9 Immunohistochemistry and multiplex immunofluorescence

A separate case series of 15 FFPE ameloblastoma specimens underwent immunohistochemical validation. Tissue sections, each 4 μm thick, were prepared and deparaffinized before undergoing heat-induced antigen retrieval. Following a blocking step, the sections were incubated overnight at 4°C with specific primary antibodies, as outlined in **Table 2**. This was followed by incubation with a secondary antibody. The signal was developed using DAB, and the slides were counterstained with haematoxylin. The evaluation of the immunostaining concentrated on identifying the precise localization of positive signals. This analysis recorded the histopathological region where distinct positive staining appeared. For quantitative analysis, digital images were evaluated using Fiji (ImageJ) software. Given its extracellular matrix deposition, FN1 expression was quantified as the positive Area% within the entire field of view. MMP9 expression was evaluated using the H-score system assisted by the IHC Profiler plugin (25), calculated as: 3*(% High Positive) + 2 *(% Positive) + 1*(% Low Positive). For paired IHC data, the Paired Student’s t-test was used for normally distributed data (MMP9), while the Wilcoxon matched-pairs signed rank test was applied for non-normally distributed data (FN1).

**Table 2:** Antibodies used for IHC and mIF.

Target antigen	Host species; clonality	Clone ID	Manufacturer	Catalog number	Dilution
FN1	Rabbit; Monoclonal	[ARC61164]	Abclonal	A23830	1:500
MMP9	Rabbit; Polyclonal	N/A	Abclonal	A0289	1:100
CTSK	Rabbit; Polyclonal	N/A	Bioss	bs-1611R	1:100

To definitively attribute the cellular source and validate spatial relationships at single-cell resolution, multiplex immunofluorescence was performed on 5 independent validation cases. FFPE sections were sequentially stained using a multiplex IHC kit according to the manufacturer’s protocol. The primary antibodies included specific TAC-OGE lineage markers (CTSK), FN1, and MMP9. Nuclei were counterstained with DAPI. Fluorescence images were captured and spatial colocalization was quantitatively analysed using line intensity profiling.

## 4 Result

### 4.1 Quality control and spatial transcriptomic landscape of ameloblastoma

**Figure 1:**
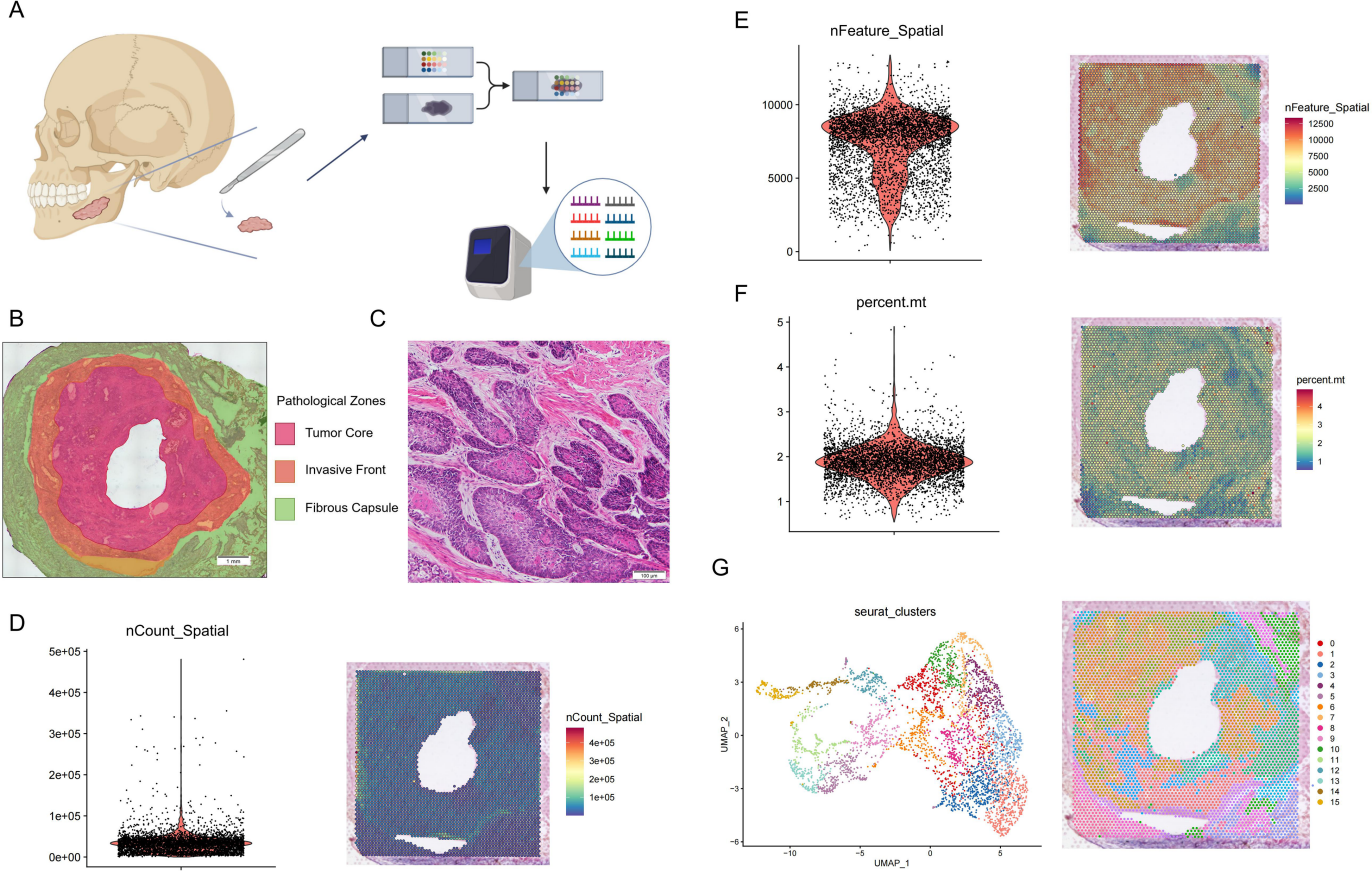
Spatial transcriptomic profiling and architectural mapping of ameloblastoma. **(A)** Schematic diagram illustrating the experimental workflow for spatial transcriptomic profiling. Created in BioRender. Wang, Y. (2026). https://biorender.com/8dz7kwn. **(B)** Histopathological zoning of the specific ameloblastoma tissue section subjected to spatial transcriptomic analysis. **(C)** A high-magnification H&E image from another representative section of the same patient. **(D-F)** Quality control metrics for the spatial transcriptomics libraries. **(D)** Distribution of total reads per spot. **(E)** Number of genes detected per spot. **(F)** Mitochondrial gene percentage per spot. **(G)** Visualization of the cellular heterogeneity and spatial distribution. (Left) UMAP plot showing the clustering of spots based on gene expression profiles, with colours representing distinct clusters. (Right) Spatial mapping of the identified clusters back onto the original tissue architecture, demonstrating the topographic distribution of different transcriptional domains.

To unravel the spatial molecular architecture of ameloblastoma, we utilised 10x Visium spatial transcriptomics on a fresh-frozen human mandibular ameloblastoma specimen (**Figure 1A**). Post-sequencing and processing, we captured 4377 high-quality tissue spots for detailed analysis. Quality control metrics validated the robustness of our data, with gene detection per spot and total UMI counts displaying anticipated distributions across the tissue section (**Figures 1D–F**). Spatial clustering of spots, based on gene expression profiles, effectively mirrored the histological features of the tumour, including the tumour core, fibrous capsule, and invasive front (**Figure 1G**). Histological annotations by an expert pathologist were overlaid to corroborate the spatial clustering assignments (**Figure 1B**).

### 4.2 Decoding cellular heterogeneity and annotating tumour microenvironment clusters

**Figure 2:**
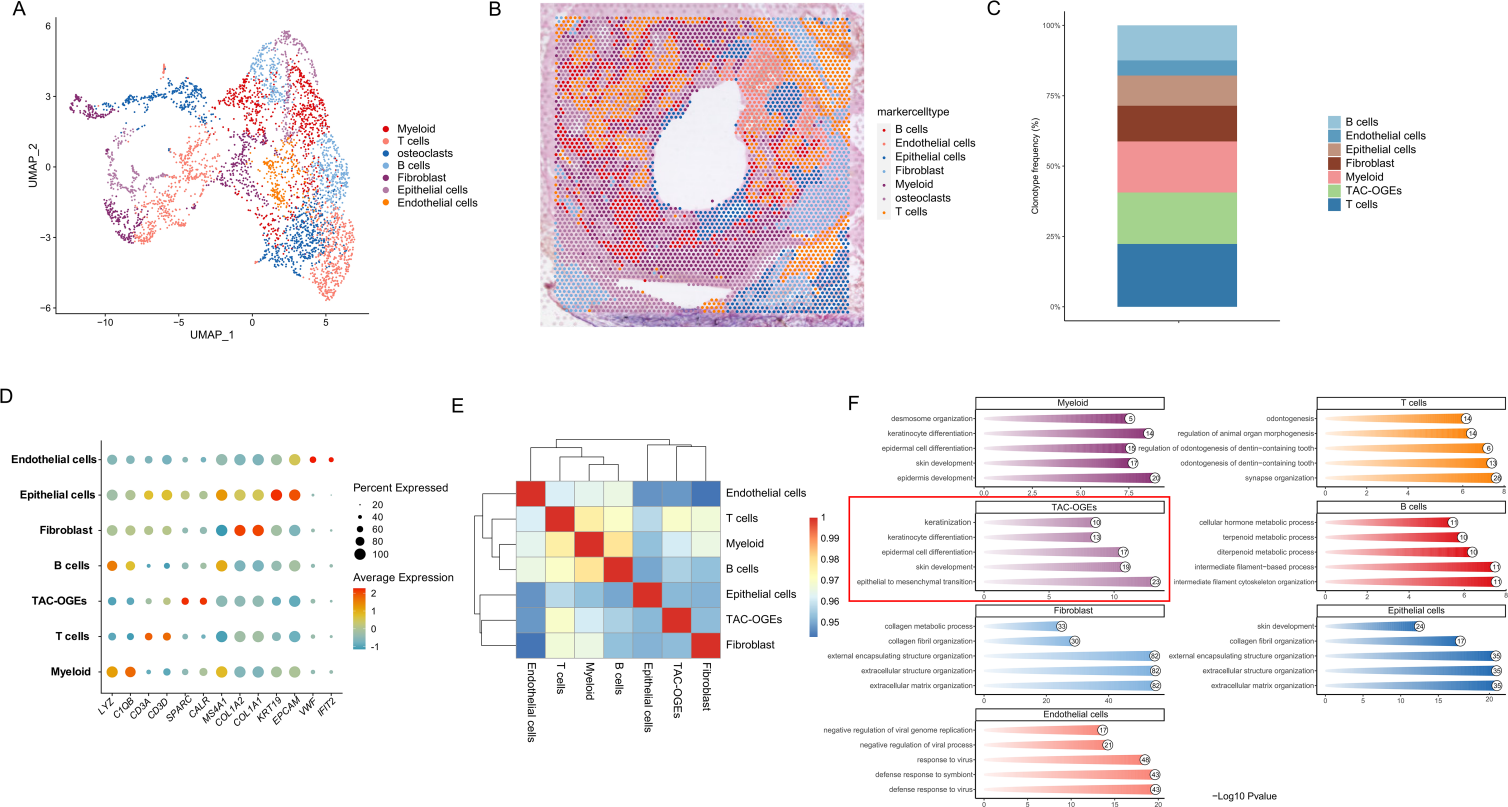
Identification and spatial distribution of distinct cell populations. **(A)** UMAP visualization of all spots, coloured by the annotated major cell types. **(B)** Spatial distribution of the annotated cell types mapped back onto the tissue section. **(C)** Bar plot showing the proportion (%) of each annotated cell type across the entire dataset. **(D)** Heatmap displaying the expression of canonical marker genes for each identified cell cluster. Rows represent genes, columns represent clusters. **(E)** Correlation heatmap depicting the pairwise transcriptional similarity between different cell clusters. **(F)** Results of Gene Ontology (GO) biological process enrichment analysis for the differentially expressed genes of each cell cluster.

Through unsupervised clustering of spatial transcriptomics data, we identified seven distinct cell populations, each residing in specific spatial niches within the tumour microenvironment (**Figures 2A, B**). Using the expression profiles of canonical marker genes, we accurately annotated these clusters (**Figure 2D**):

Epithelial tumour cells are distinguished by their elevated expression of KRT19 and EPCAM.

Fibroblasts: Enriched for collagen genes COL1A1 and COL1A2.

Myeloid Cells: Marked by LYZ and C1QB.

Endothelial Cells: Expressing VWF and PECAM1.

T Cells: Identified by CD3D and CD3E.

B Cells: Expressing CD79A and MS4A1.

Osteoclasts form a distinctive cluster characterised by the expression of CALR, SPARC, and CTSK.

Spatial mapping analysis revealed that the cell cluster initially annotated as osteoclasts exhibited a highly specific spatial distribution, being almost exclusively enriched at the invasive front of the tumour. This unique spatial localization strongly suggests that this cell population may be closely associated with the local invasive behaviour of ameloblastoma, warranting further in-depth investigation.

To further explore their role at the invasive front, we designated this cluster as the primary focus of our subsequent analyses. However, upon retrospective examination of the corresponding H&E-stained tissue sections, we observed that the histological structures underlying these transcriptomically defined osteoclast spots lacked typical osteoclast morphology; specifically, no distinct multinucleated giant cells or bone resorption lacunae were identified. Integrating these morphological observations with their robust expression of osteoclast-related genes, we redefined this population more precisely as ‘Tumour-associated cells with osteoclast-like gene expression’ (TAC-OGEs). This term accurately describes a distinct tumour-associated cell population displaying an osteoclast-like transcriptomic signature, fundamentally distinct from classical bone-resorbing osteoclasts.

To acquire a more profound understanding of each cluster’s function, we conducted GO and KEGG pathway enrichment analyses on the differentially expressed genes within each cluster (**Figure 2F**, **Supplementary Figure 1**).

### 4.3 Intercellular communication highlights pro-invasive signalling from TAC-OGEs

**Figure 3:**
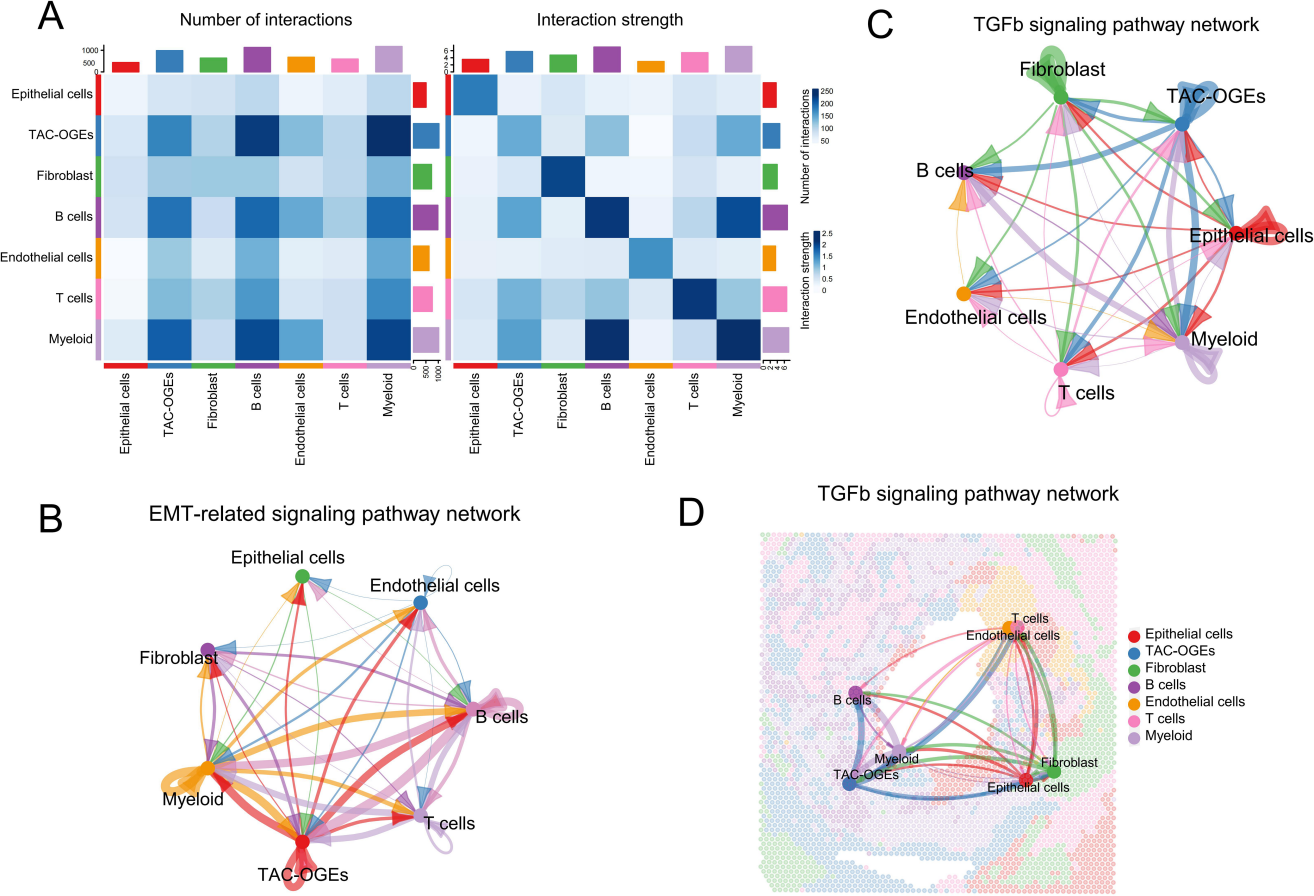
Cell-cell communication networks suggest potential pro-invasive signalling at the tumour-stroma interface. **(A)** Global assessment of cell-cell communication. (Left) The number of inferred interactions between each pair of cell clusters. (Right) The overall communication strength among cell clusters. Both diagrams summarize the total outgoing and incoming signalling across the identified cell types. **(B)** Specific analysis of epithelial-mesenchymal transition (EMT)-related signalling. The network plot depicts the inferred cell-cell communication network associated with EMT-related pathways among cell clusters, highlighting potential niches regulating EMT processes. **(C, D)** Detailed dissection of TGF-β signalling.

To elucidate the potential crosstalk within the ameloblastoma microenvironment, we inferred cell-cell communication networks. Our findings identified TAC-OGEs as prominent senders and receivers of pro-tumourigenic signals (**Figure 3A**). Notably, these interactions showed significant enrichment in pathways associated with ECM reorganisation and EMT (**Figures 3B–D**). This suggests a model in which signals from TAC-OGEs may participate in remodelling the surrounding matrix, which is associated with a pro-invasive tumour cell phenotype.

### 4.4 Pseudotime trajectory unravels a spatially ordered TAC-OGEs differentiation continuum

**Figure 4:**
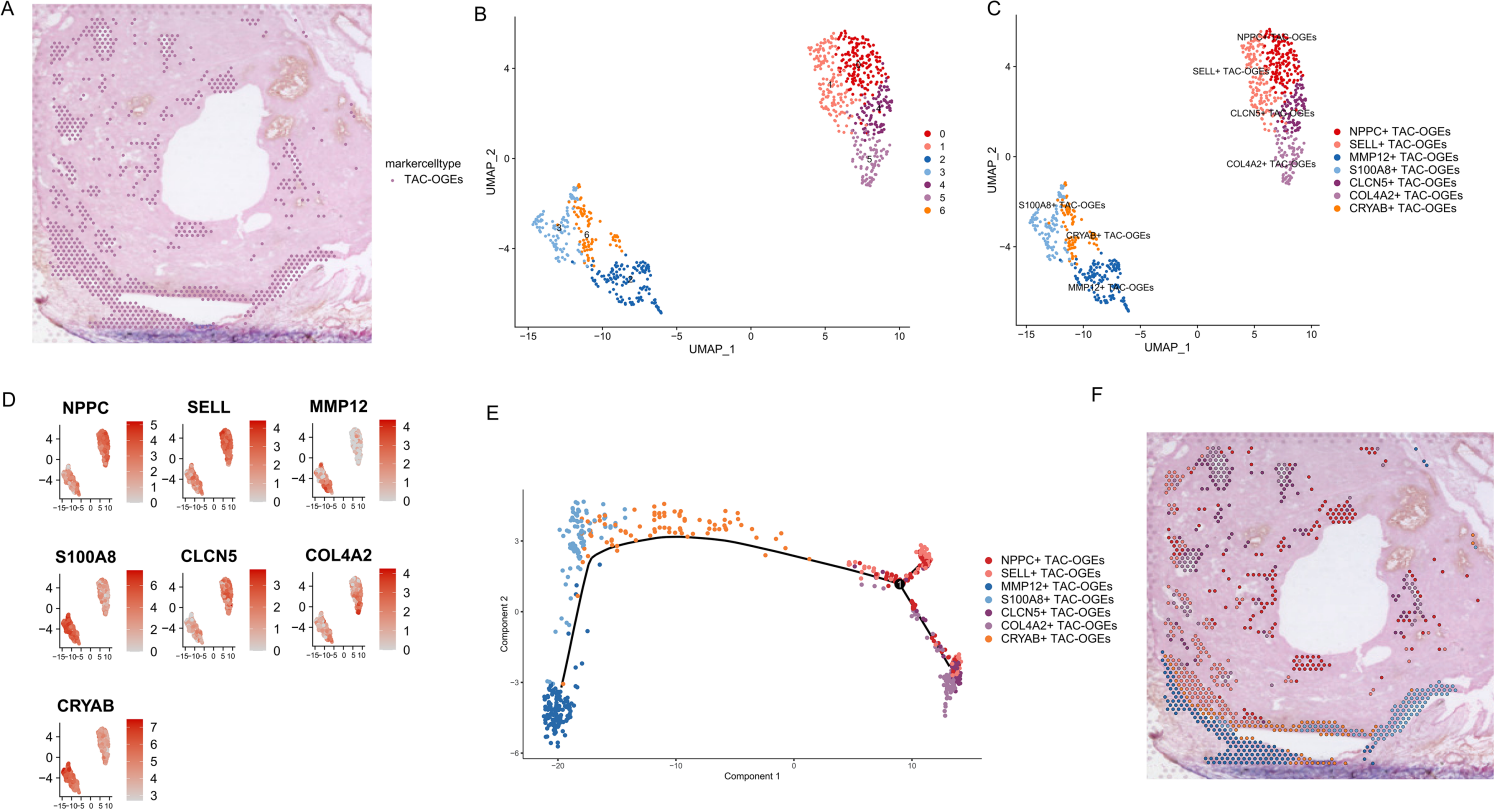
Transcriptional heterogeneity and spatially ordered phenotypic transition of TAC-OGEs. **(A)** Spatial localization of TAC-OGEs. **(B)** Re-clustering of TAC-OGEs spots. UMAP visualization of all spots identified as TAC-OGEs, revealing intrinsic transcriptomic heterogeneity within this population. **(C)** Annotation of TAC-OGEs subclusters. The UMAP from **(B)** is coloured by the newly identified TAC-OGEs subclusters. **(D)** Marker gene expression for TAC-OGEs subclusters. **(E)** Pseudotime trajectory analysis of TAC-OGE subclusters. The plot displays the inferred developmental trajectory overlaid on cells coloured by their respective subclusters, suggesting a potential differentiation or activation continuum from COL4A2+ TAC-OGEs to MMP12+ TAC-OGEs. **(F)** Spatial mapping of the trajectory states. The subclusters corresponding to different inferred developmental stages from **(E)** are projected back onto the tissue architecture, visualizing their spatial distribution.

Given their pivotal role in communication, our focus was directed towards the TAC-OGE population. Through re-clustering, we identified seven distinct TAC-OGEs subclusters, each characterised by unique transcriptional profiles, marked by high expression levels of NPPC, SELL, MMP12, S100A8, CLCN5, COL4A2, and CRYAB, respectively (**Figures 4A–D**). Pseudotemporal ordering of these subclusters revealed a continuous differentiation trajectory. Notably, this pseudotime axis exhibited a strong correlation with the spatial distribution of the cells: the trajectory commenced from precursor-like states (COL4A2+) located in the inner tumour and culminated with the mature, ECM-remodelling MMP12+ subcluster, which was exclusively situated at the invasive front of the tumour (**Figures 4E, F**). Rather than a classic lineage differentiation, this trajectory likely represents a spatial phenotypic transition of TAC-OGEs. The progression from a COL4A2+ state (associated with structural basement membrane/matrix interaction) to an actively degrading MMP12+ state reflects the dynamic functional adaptation of these cells at the invasive margin. To elucidate the functional divergence along this trajectory, we performed Gene Ontology and Kyoto Encyclopedia of Genes and Genomes pathway enrichment analyses for each subcluster (**Supplementary Figures 2**, **3**).

### 4.5 Co-upregulation of FN1 and MMP9 at the invasion front and proteomic validation

**Figure 5:**
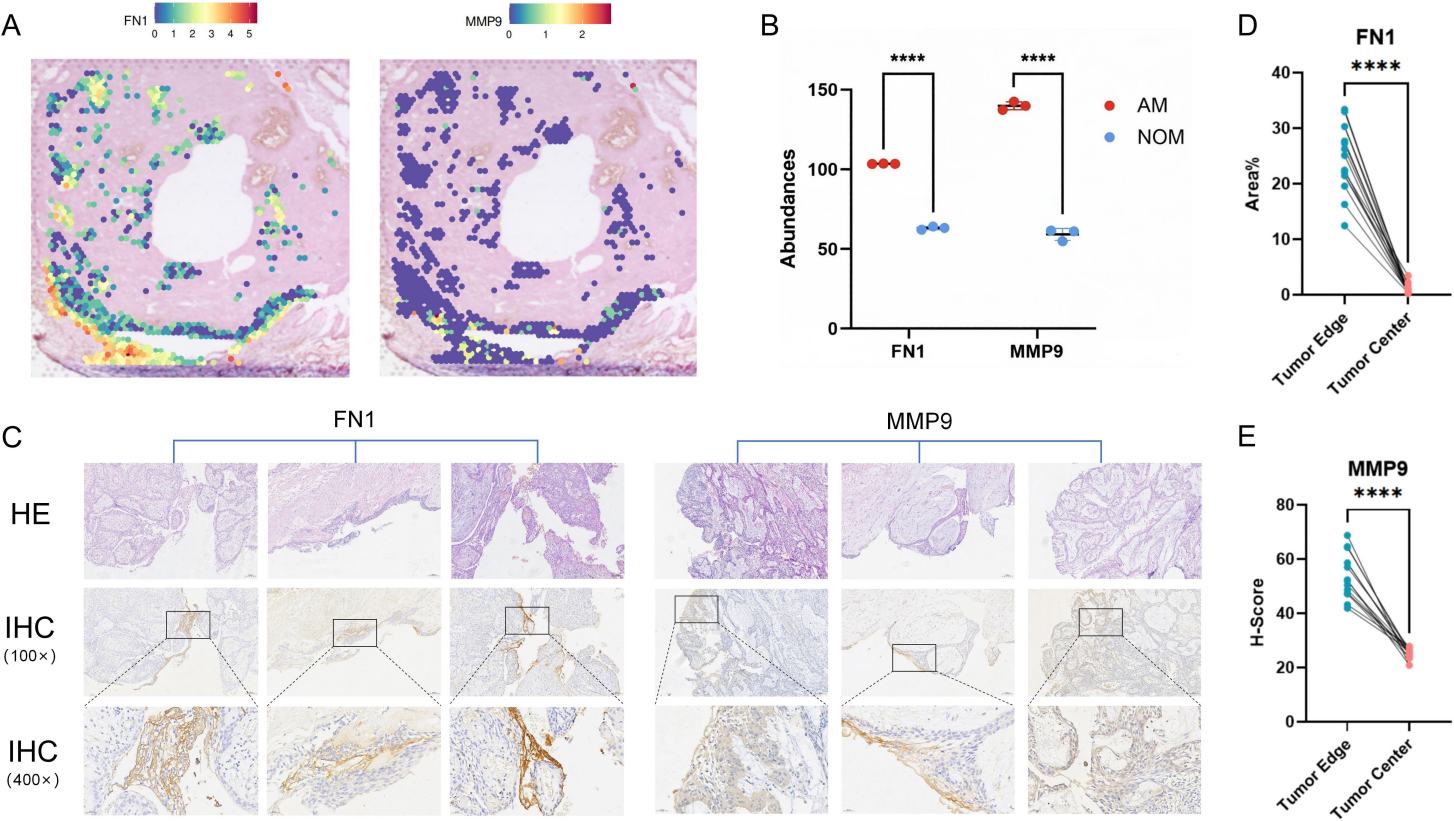
Spatial transcriptomic and proteomic validation of FN1 and MMP9 enrichment at the invasive front. **(A)** Spatial distribution of FN1 and MMP9 expression. Representative spatial feature plots show the transcriptomic expression patterns of FN1 (Left) and MMP9 (Right) mapped onto the tissue architecture. **(B)** Differential expression of FN1 and MMP9 in ameloblastoma versus normal tissue. A scatter dot plot comparing the average expression levels of FN1 and MMP9 between ameloblastoma samples (n=3) and matched normal tissue controls (n=3). Data are analysed by two-tailed unpaired Student’s t-test. **(C)** Immunohistochemical (IHC) validation of FN1 and MMP9 protein expression in ameloblastoma. **(D, E)** IHC data are presented as paired dot plots.****P < 0.0001 (by Paired t-test for MMP9; Wilcoxon matched-pairs signed rank test for FN1).

We explored the molecular mechanisms driving invasion within the high-risk MMP12+ TAC-OGEs. Our analysis revealed that FN1 and MMP9 are significantly upregulated in this subcluster compared to others (**Figure 5A**). The elevated expression of these ECM-remodelling molecules is correlated with the potential for local invasion. To confirm the clinical significance of these findings, we utilised an existing proteomics dataset. In alignment with our spatial transcriptomic data, protein levels of FN1 and MMP9 were markedly higher in ameloblastoma tissue samples than in healthy gingival control tissues (**Figure 5B**).

### 4.6 Immunohistochemical and multiplex immunofluorescence validation reveals enrichment of FN1 and MMP9 at the tumour-stroma interface

Immunohistochemical analysis on a separate case series (n=15) verified the unique spatial expression of FN1 and MMP9 proteins. Both markers were predominantly concentrated at the invasive tumour margin. FN1 exhibited significant extracellular matrix deposition, whereas MMP9 was mainly expressed in cells at the advancing edge (**Figures 5C–E**). These findings visually support the molecular characteristics identified at the tumour-stroma interface via spatial transcriptomics.

**Figure 6:**
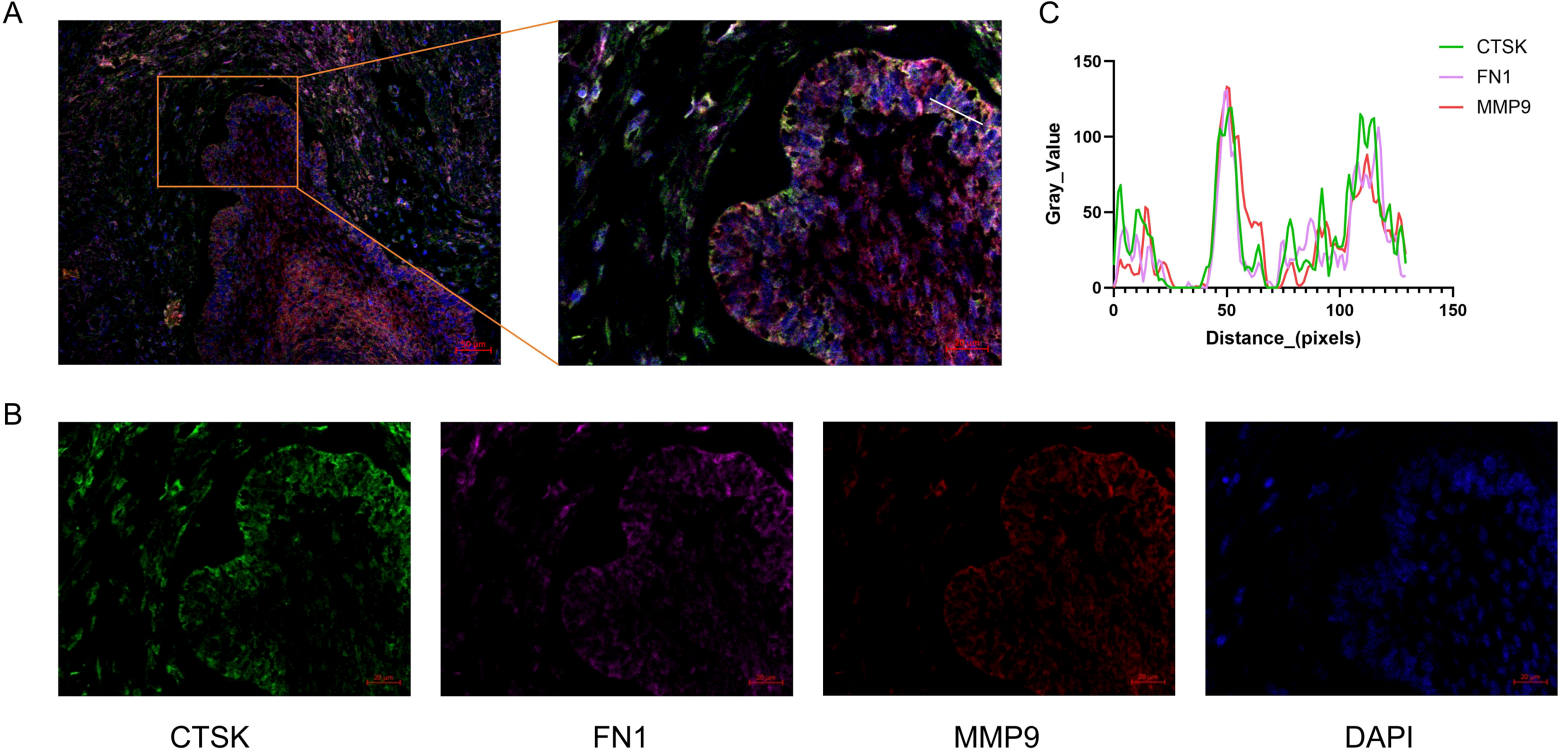
Multiplex immunofluorescence confirms the spatial co-localization of TAC-OGEs with FN1 and MMP9. **(A)** Representative merged mIF image illustrating the spatial architecture of the tumour ecosystem. The boxed region is magnified in the right panel to specifically highlight the tumour-stroma interface. **(B)** Single-channel fluorescence images resolving the specific spatial distribution of CTSK (green, serving as the marker for the TAC-OGE population), FN1 (magenta, extracellular matrix deposition), MMP9 (red, matrix-degrading enzyme), and DAPI (blue, cell nuclei). The images explicitly show the enrichment of CTSK, FN1, and MMP9 at the advancing margin. **(C)** Quantitative spatial co-localization analysis via a line intensity profile. The graph plots the fluorescence intensity (Gray Value) across the physical distance (pixels) corresponding to the white line drawn in the magnified panel of **(A)**. The highly synchronized, overlapping peaks of CTSK (green), FN1 (magenta), and MMP9 (red) demonstrate their robust spatial co-localization at the invasive niche.

Crucially, the mIF results explicitly demonstrated a striking spatial co-localization of TAC-OGE lineage markers with FN1 and MMP9, supporting that these molecules are co-localized within the TAC-OGE-enriched niche (**Figure 6**).

## 5 Discussion

This study utilised spatial transcriptomics to create a high-resolution molecular and cellular atlas of ameloblastoma. By employing canonical marker genes for cell type annotation, the research elucidated the complex heterogeneity within the tumour microenvironment. Notably, the group identified as the ‘TAC-OGE cluster’ through transcriptional profiles does not correspond to fully differentiated, bone-resorbing osteoclasts. Instead, it comprises a distinct subset of tumour-associated cells displaying a prominent osteoclast-like transcriptional signature. Given that our analysis is based on correlative spatial transcriptomic and histological data, these findings should be interpreted as associations rather than direct evidence of causality. Our study therefore provides a spatial and transcriptional framework that will inform future functional validation.

Building upon this transcriptional distinction, it is crucial to recognize that unlike the malignant tumour microenvironment, which is predominantly orchestrated for systemic dissemination and distant metastasis, the TME of benign yet locally aggressive neoplasms is uniquely specialized for overcoming localized physical barriers. In locally destructive lesions such as ameloblastoma and giant cell tumour of bone, non-metastatic neoplastic cells actively hijack and reprogram host stromal and monocytic cells to facilitate aggressive matrix degradation and bone resorption (26, 27). Within this framework, we redefined the actively remodelling niche at the invasive front as ‘Tumour-associated cells with osteoclast-like gene expression’ (TAC-OGEs). These cells are not merely classical physiological osteoclasts, but a highly plastic, tumour-reprogrammed population.

Having established the identity of TAC-OGEs, our analysis revealed their highly specific spatial orientation. This TAC-OGE cluster was distinctly localised at the invasive front of the tumour. It was characterised by the strong co-expression of two pro-invasive molecules, FN1 and MMP9. IHC and mIF independently validated this precise co-expression pattern in different patient case series, confirming the significant enrichment of FN1 and MMP9 proteins specifically at the tumour-stroma interface. The simultaneous upregulation of these effector molecules within the TAC-OGEs suggests a potential synergistic association with stromal remodelling and local tumour progression.

Mechanistically, FN1, a key mediator of cell–matrix adhesion, is a recognised facilitator of metastatic spread in various carcinomas. In hepatocellular carcinoma and breast cancer, FN1 promotes tumour invasion by interacting with integrin receptors and triggering downstream FAK/AKT and FAK/Src signalling pathways (28, 29). Our spatial analysis revealed that FN1 deposition precisely aligns with MMP9-rich areas at the invasive front, consistent with ECM degradation. This spatial association suggests a model in which FN1 is co-localized and deposited within the TAC-OGE-enriched putative invasive niche, potentially co-produced by TAC-OGEs and adjacent stromal cells, which may serve dual roles: it provides a structural scaffold for cell adhesion and migration, and acts as a proteolytic substrate for MMP9. Our findings corroborate and expand the established paradigm in osteotropic cancers, such as bone-metastatic breast and prostate cancers, where osteoclasts are known to remodel the local ECM, creating a conducive environment for tumour colonisation and growth (12, 30).

Beyond their spatial enrichment, TAC-OGEs are not a static population. Alongside the spatial analysis, we conducted a pseudo-temporal trajectory analysis to elucidate the differentiation dynamics within the TAC-OGEs. This investigation uncovered a dynamic continuum, highlighting the transition of these cells from an initial state, marked by the expression of matrix components such as COL4A2, to a terminal phenotype characterised by elevated expression of matrix-degrading enzymes like MMP12. This trajectory indicates a process of functional diversification, suggesting that TAC-OGEs may gradually adopt a more aggressive, proteolytic profile as tumour progression advances.

Our observations of transcriptional and functional adaptability find resonance in studies of localized pathological osteolytic niches. The emergence of specialized cellular subsets primed for specific roles in tissue remodelling underscores a dynamic and adaptable cellular ecosystem within these lesions. This functional adaptation is likely shaped by continuous crosstalk within the TME. To further understand intercellular communication, we utilised CellChat analysis, which highlighted significant signalling interactions between the TAC-OGEs and other clusters. These CellChat inferences are hypothesis-generating and suggest that TGF-β and IL-1 pathways might act as potential mediators of this process, requiring future experimental validation. Our data thus align with the established mechanisms of localized bone destruction, where continuous epithelial-mesenchymal crosstalk, in which osteoclast activation is associated with subsequent tissue remodelling and invasion.

Crucially, to determine whether this TAC-OGE-mediated invasive mechanism is a universal feature of ameloblastoma, we evaluated its presence across different clinical variants. According to the latest WHO classification (31), our case series encompassed 11 conventional and 4 unicystic ameloblastomas. While the limited sample sizes of these specific subgroups preclude robust statistical correlation analyses between overall FN1/MMP9 expression levels and individual histological subtypes or clinical outcomes, our qualitative evaluation of both the IHC and the multiplex immunofluorescence data revealed a highly conserved spatial pattern. Specifically, the pronounced enrichment of FN1 and MMP9 at the TAC-OGE-mediated tumour-stroma interface was consistently observed across both conventional and unicystic cases. This suggests that TAC-OGE-associated marginal matrix remodelling may represent a fundamental, conserved feature correlated with local invasion in ameloblastoma, regardless of the internal histological architecture.

The identification of this conserved, highly active niche presents new therapeutic vulnerabilities. Our findings suggest that the TAC-OGEs may represent a potential therapeutic target for ameloblastoma treatment. Clinically approved agents, such as bisphosphonates and the RANKL inhibitor denosumab, are known to effectively suppress osteoclast activity. These agents have shown success in managing osteolytic complications in malignant bone diseases, including bone metastases from breast cancer and multiple myeloma (32, 33). While anti-resorptive agents like denosumab present a compelling clinical rationale, their efficacy hinges on the assumption that TAC-OGEs utilize canonical RANKL-dependent pathways. If TAC-OGEs operate independently of RANKL, alternative matrix-targeting strategies must be considered. Future studies mapping the specific dependency of TAC-OGEs on the RANK/RANKL axis are essential before clinical repurposing can be validated.

The heterogeneity and dynamic differentiation continuum observed within the TAC-OGEs, particularly the shift from a COL4A2-expressing to an MMP12-high phenotype, presents opportunities for therapeutic advancement. By targeting specific transitional subpopulations or the signalling pathways responsible for this phenotypic change, treatment precision could be improved, potentially circumventing compensatory mechanisms. Future research should focus on directly correlating these molecularly defined TAC-OGE subsets with clinical parameters, such as recurrence rates, radiographic invasion patterns, or surgical margin status. Such analyses are essential for refining risk stratification models and providing a molecular basis for identifying patients who would benefit most from adjuvant anti-osteoclast therapy.

Furthermore, therapeutic strategies must consider the underlying genetic drivers and cellular heterogeneity. While our study highlights the role of TAC-OGEs in ameloblastoma invasion, it is important to consider these findings within the broader context of known genetic drivers, such as the frequent BRAF V600E mutation. Previous studies have suggested a link between BRAF mutation status and the upregulation of pro-invasive factors like MMP9 (34); however, the precise cross-talk between BRAF-mutated epithelial cells and the TAC-OGE-mediated invasive niche remains to be fully elucidated. From a therapeutic standpoint, targeting the microenvironment may offer complementary clinical benefits. While BRAF/MEK inhibitors effectively target the intrinsic proliferative drive of the epithelial tumour cells, therapies directed against TAC-OGEs may potentially modulate microenvironmental processes associated with local tissue destruction. We hypothesize that a combination approach could potentially provide a synergistic effect—limiting both tumour growth and physical invasion. Furthermore, targeting this conserved spatial mechanism of marginal invasion could represent a potential therapeutic alternative for patients with BRAF wild-type tumours. Future functional studies are required to validate these proposed combined interventions.

While our integrated spatial omics approach offers a novel perspective, several inherent limitations must be acknowledged. Primarily, the spatial transcriptomic profiling was conducted on a single, albeit representative, tumour specimen. This allowed for an in-depth proof-of-concept analysis but restricts the evaluation of inter-tumour heterogeneity and the generalisability of the identified cellular states, especially regarding the spectrum of TAC-OGEs differentiation. Consequently, validation across larger, multi-institutional cohorts is crucial to confirm the prevalence and consistency of these molecularly defined TAC-OGE subpopulations. Secondly, our study’s cross-sectional design does not include longitudinal sampling from the same patients. As a result, we are unable to make definitive conclusions about the temporal dynamics and evolution of TAC-OGEs phenotypes, particularly concerning key clinical events like disease progression, recurrence, or therapeutic response. Future research involving serial biopsies or patient-derived xenograft models would be crucial in addressing this limitation.

Additionally, technical constraints influenced our control group selection and functional validation. Ideally, the developing dental lamina serves as the perfect biological control for ameloblastoma; however, due to strict ethical and practical limitations in obtaining human embryonic dental tissues, adjacent healthy gingival tissue was utilized as the control in our proteomic comparisons. Given that ameloblastomas can arise from the basal cells of the oral epithelium, and both share a common ectodermal origin, gingival tissue represents a widely accepted and feasible alternative for comparative omics in odontogenic research (35).

Finally, although our correlative spatial and IHC data indicate a co-expression of FN1 and MMP9 within the TAC-OGEs, they fall short of demonstrating direct causality. To conclusively establish that TAC-OGE-derived FN1 and MMP9 drive ameloblastoma invasion, targeted functional experiments are essential. These should employ knockdown or knockout techniques in relevant *in vitro* co-culture systems or genetically engineered *in vivo* models, which will also help elucidate the specific signalling pathways involved.

In summary, this study combines spatial transcriptomics with immunohistochemical and multiplex immunofluorescence validation to systematically chart the cellular and molecular landscape of ameloblastoma. We identify a unique TAC-OGE cluster, spatially confined to the invasive front, distinguished by the co-expression of FN1 and MMP9. These insights offer a novel spatial framework linking stromal remodeling by TAC-OGEs with the local aggressive behaviour of ameloblastoma.

## Data Availability

The datasets presented in this study can be found in online repositories. The names of the repository/repositories and accession number(s) can be found below: https://ngdc.cncb.ac.cn, accession number OMIX014153.

## Supplementary material

Supplementary Figure 1 KEGG pathway enrichment analysis for each transcriptional cluster.

Supplementary Figure 2 GO enrichment analysis of TAC-OGE subclusters.

Supplementary Figure 3 KEGG pathway enrichment analysis of TAC-OGE subclusters.

Supplementary Figure 4 Negative controls for immunohistochemistry. Representative images of negative control staining in ameloblastoma tissue sections. To confirm antibody specificity, primary antibodies were omitted and replaced with phosphate-buffered saline (PBS) during the staining procedure. All other experimental conditions, including secondary antibody incubation and chromogenic detection, remained identical to those of the experimental groups. No specific immunoreactivity was observed.

Supplementary Table 1 Quantitative proteomics data of FN1 and MMP9 expression in ameloblastoma and normal control tissues.
