# Supplementary figures and images for: Spatial transcriptomics uncovers TAC-OGEs heterogeneity and FN1/MMP9 signature in ameloblastoma
Source: Front Immunol. 2026 Apr 30;17:1770116. doi: 10.3389/fimmu.2026.1770116 (PMC13171749; doi:10.3389/fimmu.2026.1770116)

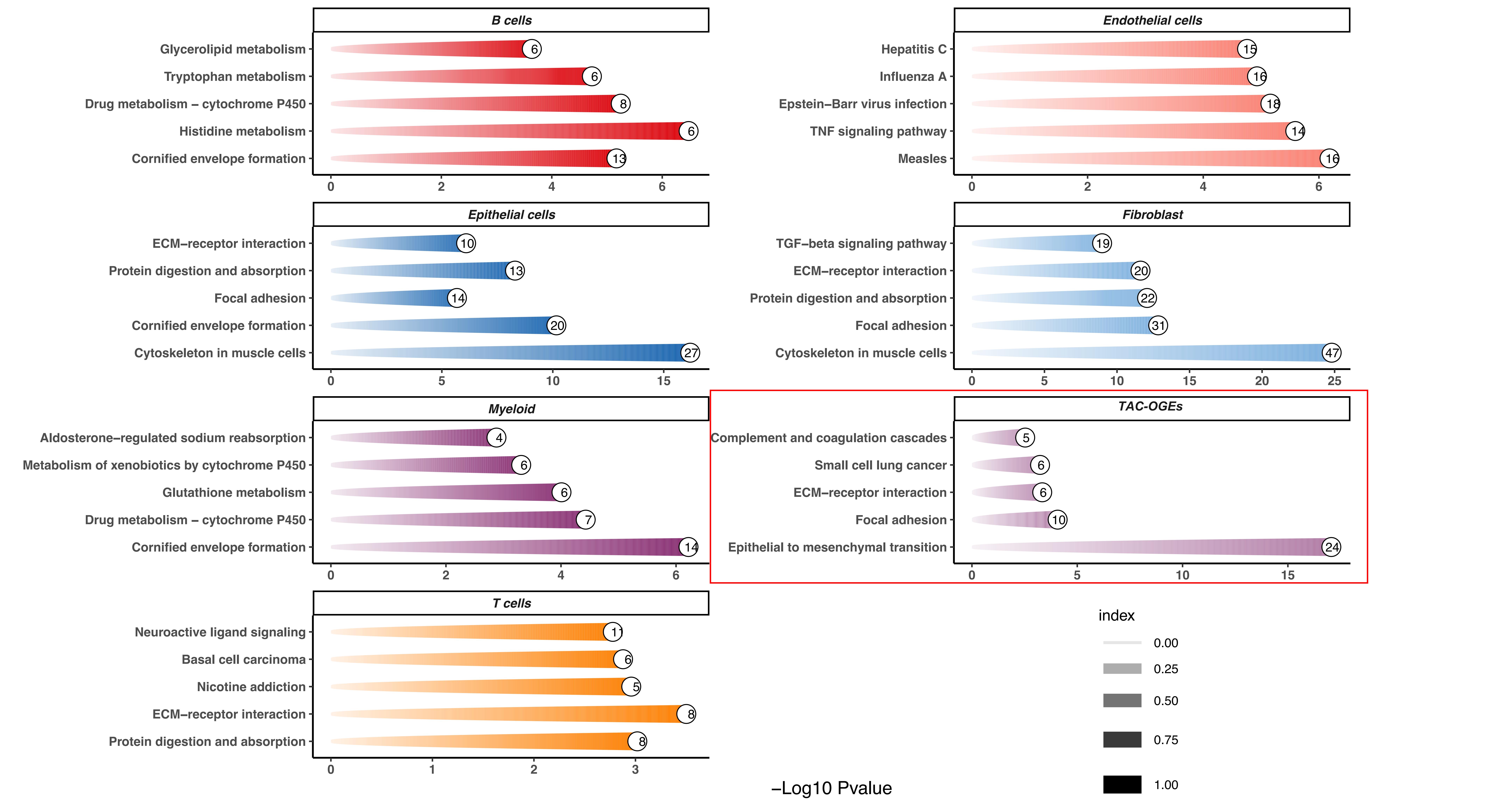

Supplement: Supplementary Figure 1 — KEGG pathway enrichment analysis for each transcriptional cluster. [file Image1.jpeg]

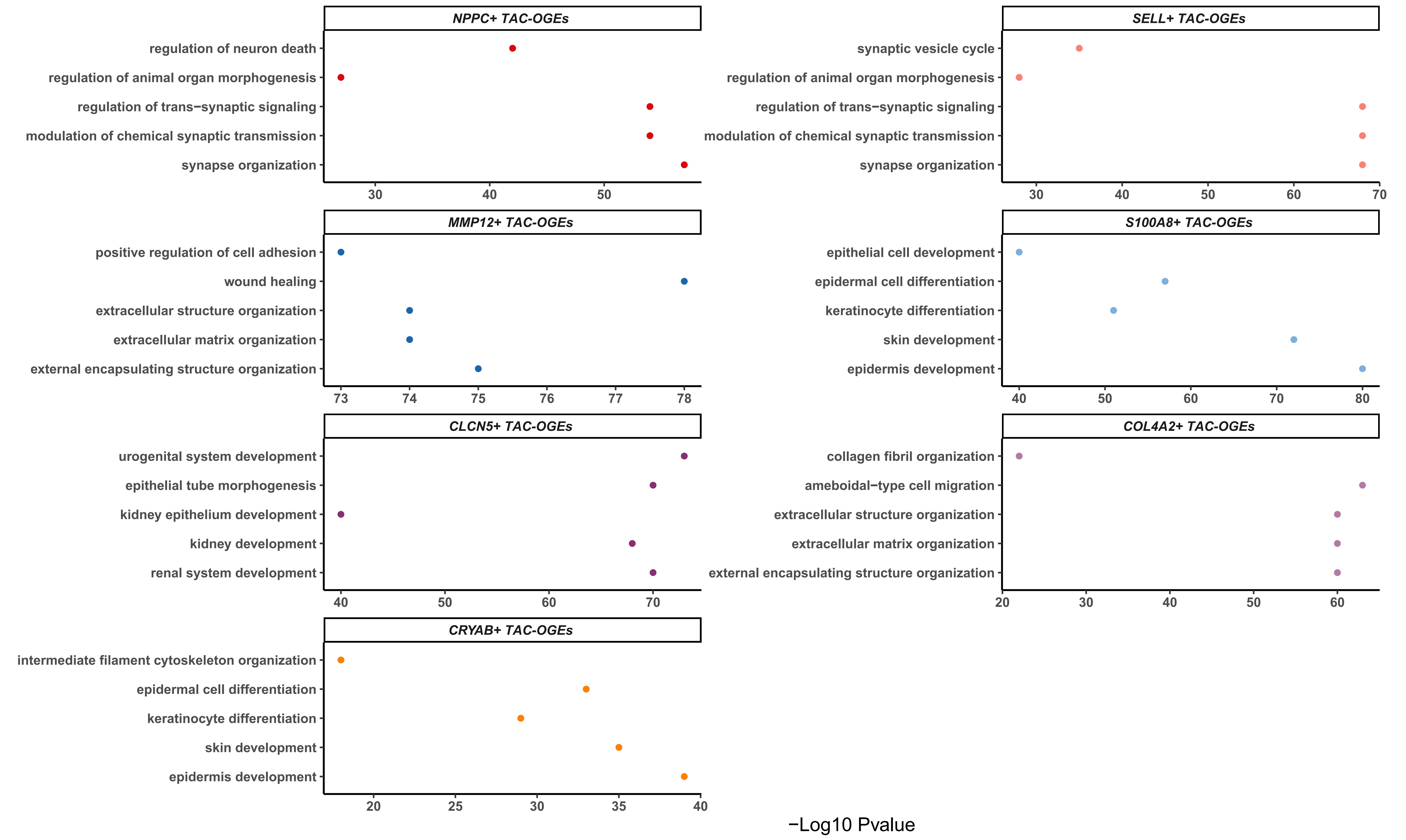

Supplement: Supplementary Figure 2 — GO enrichment analysis of TAC-OGE subclusters. [file Image2.jpeg]

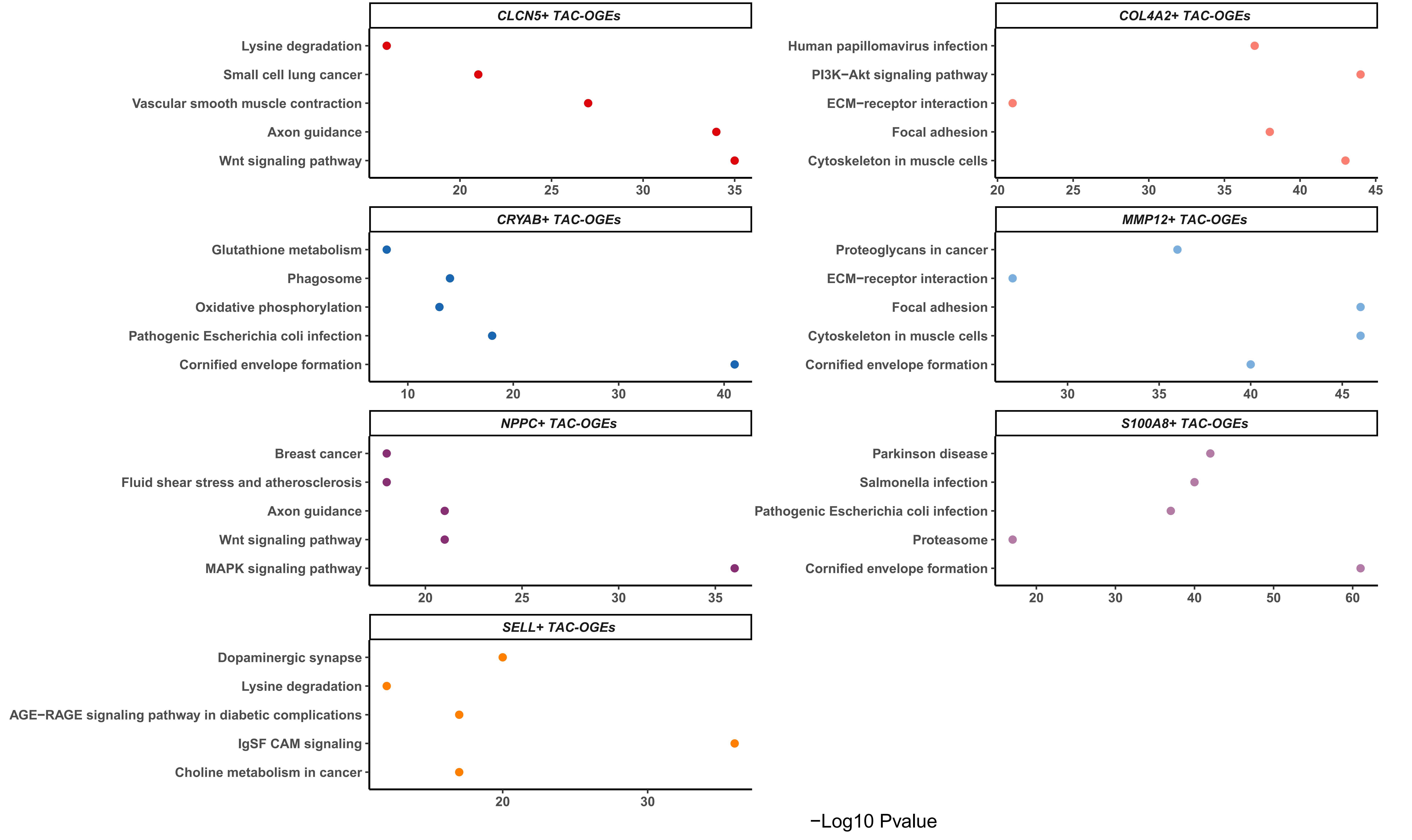

Supplement: Supplementary Figure 3 — KEGG pathway enrichment analysis of TAC-OGE subclusters. [file Image3.jpeg]

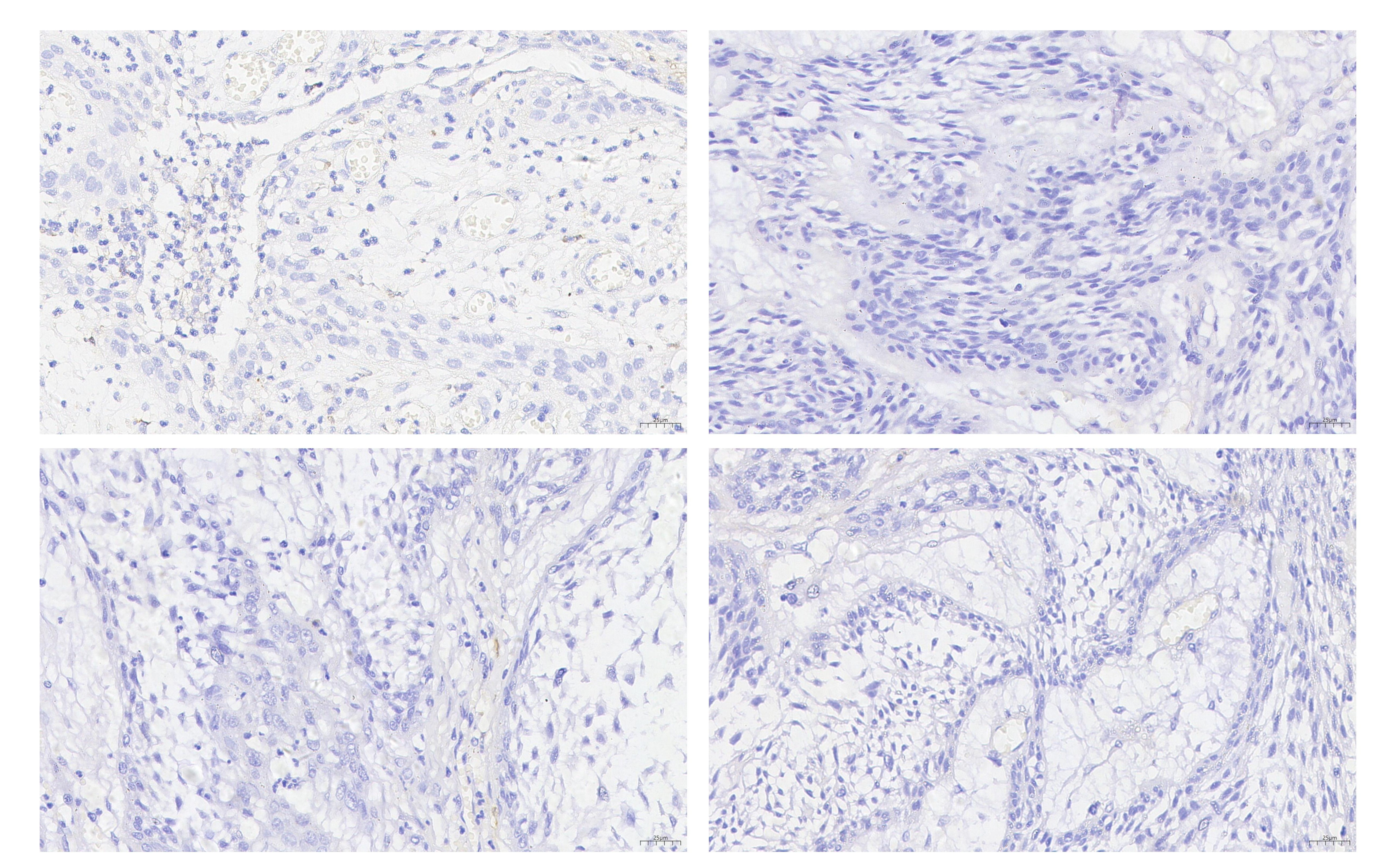

Supplement: Supplementary Figure 4 — Negative controls for immunohistochemistry. Representative images of negative control staining in ameloblastoma tissue sections. To confirm antibody specificity, primary antibodies were omitted and replaced with phosphate-buffered saline (PBS) during the staining procedure. All other experimental conditions, including secondary antibody incubation and chromogenic detection, remained identical to those of the experimental groups. No specific immunoreactivity was observed. [file Image4.jpeg]
